# The Hip Lag Sign - Prospective Blinded Trial of a New Clinical Sign to Predict Hip Abductor Damage

**DOI:** 10.1371/journal.pone.0091560

**Published:** 2014-03-12

**Authors:** Alexander Kaltenborn, Catherine M. Bourg, Andreas Gutzeit, Fabian Kalberer

**Affiliations:** 1 Department of Orthopedic Surgery, Kantonsspital Winterthur, Winterthur, Switzerland; 2 Department of Trauma and Orthopedic Surgery, Federal Armed Forces Hospital Westerstede, Westerstede, Germany; 3 Department of General, Visceral and Transplant Surgery, Hannover Medical School, Hannover, Germany; 4 Department of Radiology, Kantonsspital Winterthur, Winterthur, Switzerland; 5 Department of Radiology, Paracelsus Medical University Salzburg, Salzburg, Austria; Queensland Institute of Medical Research, Australia

## Abstract

This study introduces and validates the Hip Lag Sign, a new clinical parameter to determine hip abductor damage, which appears to be one major cause for greater trochanteric pain syndrome. 26 patients who underwent standardized MRI-examination were prospectively enrolledbetween October 2009 and March 2012. A standard physical examination of the hip was performed, including the Hip Lag Sign as it is defined for the first time in this work. Hip Lag Sign results were statistically compared toMR images, to pain levels measured with the visual analogue scale and to results of the modified Harris Hip Score as a universal and well established diagnostic tool for the hip. Chi^2^- and Mann-Whitney-U-analysis were applied. Diagnostic accuracy was tested with 2×2-table-calculations.Kappa statistics were used to analyze inter-observer variability. A positive Hip Lag Sign is significantly associated with MRI-proven hip abductor damage (p<0.001). The Hip Lag Sign has a sensitivity of 89.47% and a specificity of 96.55%. The positive and negative predictive values are 94.44%, resp. 93.33%. Its diagnostic Odds Ratio is 239.000 (p<0.001; 95%-CI: 20.031-2827.819). The number needed to diagnose was 1.16.Inter-observer consistency was 98.1% and kappa statistics for inter-observer variability were 0.911. The Hip Lag Sign is specific and sensitive, easy and fast to perform and allows a reliable assessment on the hip abductors' status, especially when there is no access to further diagnostic devices such as MRI for example due to restricted resources like in developing countries. Thus, we recommend the inclusion of the Hip Lag Sign into everyday hip examinations, especially dealing with patients suffering from greater trochanteric pain syndrome.

## Introduction

10–25% of the general populationsuffer from greater trochanteric pain syndrome, characterised by persistent pain and tenderness over the greater trochanter [1]. Greater trochanteric pain syndrome is generally underestimated and underdiagnosed, resulting in a chronic undertreatment of these patients, not to forget the social and economic fallout that comes with chronic pain and limitation of mobility. Although a great variety of causes, e.g. trochanteric bursitis, iliotibial tract friction or coxa vara, are known to be attributed to greater trochanteric pain syndrom, we place our focus on lesions of the hip abductors as one of the primary causes of pain in the lateral hip region [2]–[4].

The major hip abductors are the gluteus medius and minimus muscles which are essential for gait and balance, seeing as they ensure hip stability throughout the whole gait process [5]. For this particular reason, comparable to the rotator cuff in the shoulder, lesions of these muscles are often referred to as ‘rotator cuff tears of the hip' [6]–[8], which are currently best diagnosed using magnetic resonance imaging (MRI) [9]. But MR examination is also strongly dependent from technical dependencies and the experience of the radiologists [10]. Unfortunately, hip abductor damages are often missed by physicians or even misdiagnosed as bursitis which leads to wrong treatments and prolonged pain [11]. Unnecessary infiltrations or injections of corticosteroids increase the risk of adverse events such as infections and further harm the patient [12]. If patients with greater trochanteric pain syndrome do not respond to conservative treatment, gluteal tendon pathologies definitely represent one of the most important differential diagnoses which should not be clinically missed [2].

Considering this, the paucity of research on hip abductor damage and the lack of a reliable clinical sign are surprising. Especially in times of optimizing quality and resource management against the backdrop of struggling healthcare systems, cutting down unnecessary, expensive MRI-examinations seems to be reasonable. Moreover, physicians in rural areas or developing countries who depend on physical examinations are in need of a reliable clinical sign to safely diagnose hip abductor damages [13].

This is the reason why it is imperative to look for new diagnostic tools and why we investigate the Hip Lag Sign. Aim of this study is to clarify, if the Hip Lag Sign is a reliable parameter to clinically diagnose hip abductor damage.

## Patients and Methods

**Figure 1:**
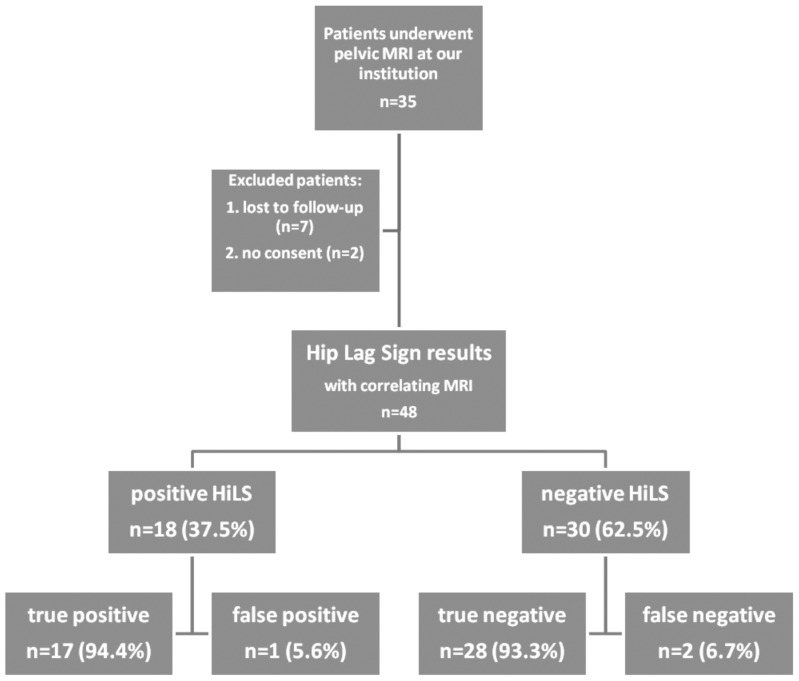
Flow Chart. Shown is the flow of patients through the Hip Lag Sign-study according to STARD-Guidelines.

This is a blinded, prospective, single-centre study with 26 patients who underwent MRI-examination at our institution between October 2009 and March 2012. MR images were statistically compared to Hip Lag Sign results, to pain levels measured with the visual analogue scale and to results of the modified Harris Hip Score as a universal and well established diagnostic tool for the hip [14], [15]. The flow of the patients through this study according to the *Standard for Reporting of Diagnostic Accuracy* (STARD) – Guidelines is pictured in Figure 1 [16].The local institutional review board approved the study protocol. Written informed consent was obtained from all patients prior to examination and inclusion.

### Inclusion and exclusion criteria

Both patients with and without total hip arthroplasty in their history were included (23 hip replacements in 16 patients [61.54%]). Excluded were cases where abductor tendon insertion was not visible on MR-imaging due to metal artefacts induced by total hip arthroplasty (n = 0), paediatric patients defined as younger than 18 years (n = 0), patients who were lost to follow-up (n = 7), and patients without informed consent (n = 2).One-sided MRI-studies were performed for 4 patients. The cases where no correlating MRI was available were excluded from statistical analysis. The participating patients' characteristics are listed in Table 1.

**Table 1:** Shown are the patients' characteristics of all included cases.

	Sex	Age	BMI	Hip Abductor Damage	Hip Lag Sign	cl. Hip Lag Sign	mHHS	VAS	THA
1	♀	69	33.7	gluteus medius PR	+	−	89	3	+
2	♀	74	28.3	gluteus medius/minimus R right & left	+	−	53	6	+
3	♀	75	23.8	ri: gluteus medius / minimus Rle: gluteus medius / minimus atrophy	+	+	52	8	+
4	♀	79	20.2	gluteus medius / minimus R	+	−	93	1	−
5	♂	49	27.9	nil	−	−	84	3	+
6	♀	78	22.0	gluteus minimus R / medius atrophy right & left	+	+	76	3	−
7	♀	62	26.4	gluteus medius / minimus atrophy	+	−	71	7	+
8	♂	57	27.8	nil	−	−	83	6	+
9	♂	60	28.1	nil	−	−	93	7	−
10	♀	27	18.4	nil	−	−	96	2	−
11	♀	48	21.4	nil	−	−	73	5	+
12	♀	74	23.9	nil	−	−	63	7	+
13	♂	78	26.9	gluteus minimus / medius PR	+	−	71	5	−
14	♀	77	24.6	gluteus medius / minimus atrophy	+	−	50	7	−
15	♀	71	25.5	gluteus medius / minimus atrophy	+	−	80	4	+
16	♀	68	28.7	gluteus minimus PR / medius tendinosis	+	−	100	6	+
17	♀	76	33.7	gluteus minimus / medius tendinosis	+	−	55	8	+
18	♀	43	38.3	nil	−	−	73	5	−
19	♀	66	28.0	gluteus minimus / medius PR	+	−	49	7	−
20	♂	24	24.2	nil	−	+	84	7	−
21	♂	80	24.7	nil	−	−	89	1	+
22	♀	75	20.6	gluteus medius atrophy	+	−	67	7	+
23	♀	76	22.7	partial gluteus medius atrophy	+	−	95	0	+
24	♂	58	24.2	gluteus minimus degeneration and PR	−	−	96	5	+
25	♀	72	24.2	gluteus medius PR	+	−	30	9	−
26	♀	66	26.2	nil	−	−	72	3	−

(BMI = Body Mass Index; Hip Lag Sign = Hip Lag Sign; cl Hip Lag Sign = contralateral Hip Lag Sign; mHHS = modified Harris Hip Score; VAS = visual analogue scale; PR = partial rupture; R = rupture).

### MR imaging

For MR imaging of the study population a 1.5 T MRI scanner (Achieva, Philips Healthcare, Best, Netherlands) was used. All MRI examinations were done in standardized supine position on the examination table and images were acquired with a 16 channel XL torso coil (Philips Healthcare, Best, Netherlands). MR imaging was used to proof hip abductor damage defined as partial or complete rupture of the gluteus minimus and/or gluteus medius tendon, tendinosis, and/or gluteus minimus/medius atrophy. MRI was analyzed by a board examined radiologist with 12 years of experience in musculoskeletal diseases.

### Physical Examination

**Figure 2:**
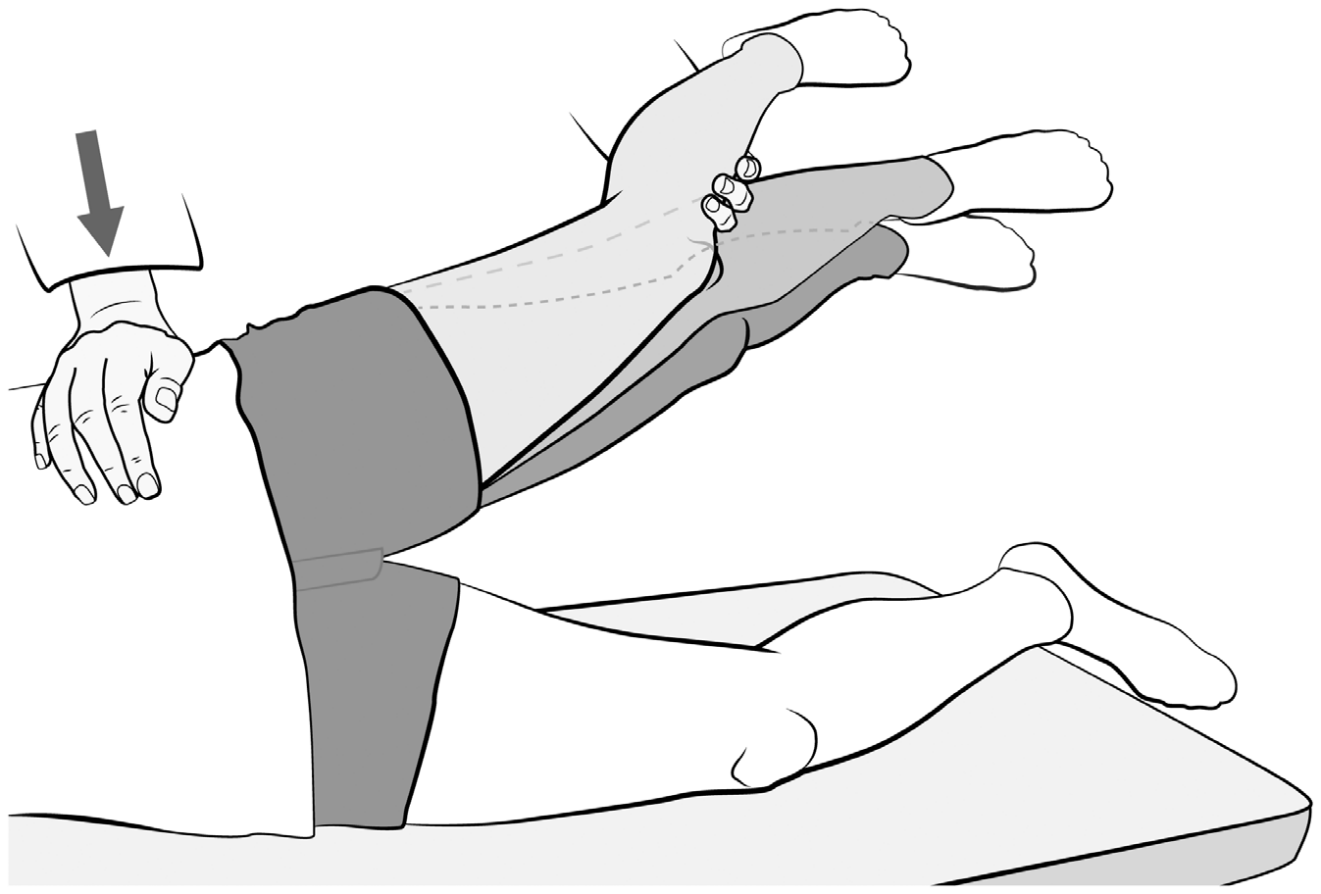
The Hip Lag Sign. Shown is the Hip Lag Sign as it is defined in this work: To test for the Hip Lag Sign, the patient has to lie in a lateral, neutral position with the affected leg being on top. The examiner then positions one arm under this leg to have good hold and control over the relaxed extremity, whereas the other hand stabilizes the pelvis. The next step is to passively extend to 10° in the hip, abduct and rotate internally as far as possible, while the knee remains in a flexed position of 45°. After the patient is asked to hold the leg actively in this position, the examiner releases the leg. The Hip Lag Sign is considered positive, if the patient is not able to keep the leg in the aforementioned abducted, internally rotated position and the foot drops more than 10 cm.

To test for the Hip Lag Sign, the patient has to lie in a lateral, neutral position with the affected leg being on top. The examiner then positions one arm under this leg to have good hold and control over the relaxed extremity, whereas the other hand stabilizes the pelvis. The next step is to passively extend to 10° in the hip, abduct to 20° and rotate internally as far as possible, while the knee remains in a flexed position of 45°. After the patient is asked to hold the leg actively in this position, the examiner releases the leg. The Hip Lag Sign is considered positive if the patient is not able to keep the leg in the aforementioned abducted, internally rotated position and the foot drops more than 10 cm. To ensure an accurate result, the test should be repeated three times (see Fig. 2).

The two clinical examiners who tested the study population for the hip lag sign were both tutored by the head of hip surgery unit at our institution immediately before the beginning of this study to allow a standardised and well executed physical examination.

They both examined the patients independently and filed their test results on an anonymous study examination sheet, which was marked with an identification number. After testing for the Hip Lag Sign on both sides, the patient was asked for her/his symptoms, any history of specific treatment or total hip arthroplasty, and a modified Harris Hip Score was evaluated. Finally the test results were compared to the MRI results of the patient.

Both examiners did not know the patient or the patient's case file beforehand. At the time of physical examination, the examiner had no knowledge which side was affected by abductor damage.

### Statistical analysis

Sensitivity, specificity and number needed to diagnose of the Hip Lag Sign were calculated using a 2×2-table (see Tabl. 2). Significance of correlations was determined with the Chi^2^-Test and the Mann-Whitney-U-Test. Furthermore, we calculated a positive and negative predictive value as well as diagnostic Odds Ratios and Likelihood Ratios according to *STARD*-guidelines [16]. Kappa statistics were used to analyze inter-observer variability.

**Table 2:** Shown is the 2×2-table to execute calculations for sensitivity, specificity, as well as positive and negative predictive value.

Hip abductor damage				
**Hip Lag Sign**		yes (n = 19)	no (n = 29)	
	positive (n = 18)	17 (94.4%)	1 (5.6%)	**PPV 94.44%**
	negative (n = 30)	2 (6.7%)	28 (93.3%)	**NPV 93.33%**
		**Sensitivity 89.47%**	**Specificity 96.55%**	

(Chi^2^-test: ***p<0.001***; Mann-Whitney-U-test: *p<0.001*).

(PPV = positive predictive value; NPV = negative predictive value).

For all statistical tests a p-value <0.05 was defined as significant. The IBM SPSS statistics software version 20.0 (IBM, Somers, NY, USA) was used to perform statistical analysis.

## Results

A total of 52 Hip Lag Signs were performed on 26 patients (7 males, 19 females; 27%, 73%, respectively) with a mean age of 64.4 years (median 71.5 years, range: 24–80 years) who fulfilled the inclusion and exclusion criteria at the time of physical examination. The average Body Mass Index defined as body weight in kg divided by height in m squared was 25.87 (median 24.65, range: 18.4–38.3).

23 of 48 examined hips (47.92%) have been treated with total hip arthroplasty, which has no statistically significant effect on the Hip Lag Sign results (p = 0.565).

There were no MR images available for four hips. Therefore, we excluded these cases and 48 Hip Lag Sign results remain for further statistical analysis. 18 Hip Lag Signs (37.5%) werepositive, which is defined as the case cohort. Thus, 30 Hip Lag Signs (62.5%) were tested negative and are representing the control group. After comparing the Hip Lag Sign results with the MR images, 17 out of 18 positive Hip Lag Signs can be confirmed true positive (94.4%) and only 2 of the 30 negative results (6.7%) must be considered as false negative (see Table 2).

Taking these results into account, we evaluated a sensitivity of 89.47% (95%-CI: 66.82%–98.39%) corresponding with a specificity of 96.55% (95%-CI: 82.17%–99.42%). The positive predictive value of the Hip Lag Sign is 94.44% (95%-CI: 72.63%–99.07%); the negative predictive value was calculated at 93.33% (95%-CI: 77.89%–98.99%). 1.16 patients have to be tested with the Hip Lag Sign to identify one case of hip abductor damage (number needed to diagnose = 1/[sensitivity-(1-specificity)] = 1.16).

A positive Hip Lag Sign result is significantly associated with MRI-proven hip abductor damage as it is defined above (p<0.001; Chi^2^- and Mann-Whitney-U-test).

As a measure of the effectiveness of a diagnostic test, the calculated diagnostic Odds Ratio is 238.000 (p<0.001; 95%-CI: 20.031–2827.819). Positive Likelihood Ratio can be reported at 25.95 (95%-CI: 3.76–179.14) and negative Likelihood Ratio at 0.11 (95%-CI: 0.03–0.41).

All but one Hip Lag Sign result were consistent between the two examiners; the non-consistent case was seen wrong positive by one physician. Thus, the inter-observer consistency is 98.1% and kappa statistics for inter-observer variability are 0.911. The Hip Lag Sign results reported in the manuscript are with the aforementioned wrong positive test included.

The mean modified Harris Hip Score in the study population is 74.04 (median: 74.50; range: 30–100). Visual analogue scale levels range from zero to nine with a mean level of 5.25 (median: 6.00).

Neither modified Harris Hip Score results (p = 0.326) nor visual analogue scale-pain level (p = 0.491) are significantly associated with positive Hip Lag Sign results. Categorization of the study population into the fourmodified Harris Hip Scoreoutcome subgroups is summarized in Table 3.

**Table 3:** Shown is the categorisation of the study population in four outcome subgroups regarding the modified Harris Hip Score.

	Modified Harris Hip Score Outcome Subgroup			
	Poor (<70)	Fair (70–79)	Good (80–89)	Excellent (90–100)
**Number of Patients**	6 (23.1%)	6 (23.1%)	6 (23.1%)	8 (30.8%)
**Abductor Damage**	4	2	3	7
**Positive Hip Lag Sign result**	3 (1 false negative)	3 (1 false positive)	3	6 (1 falsenegative)

There is no significant clustering (p = 0.195 [Chi^2^] for abductor damage; p = 0.701 [Chi^2^] for Hip Lag Sign result).

## Discussion

**Figure 3:**
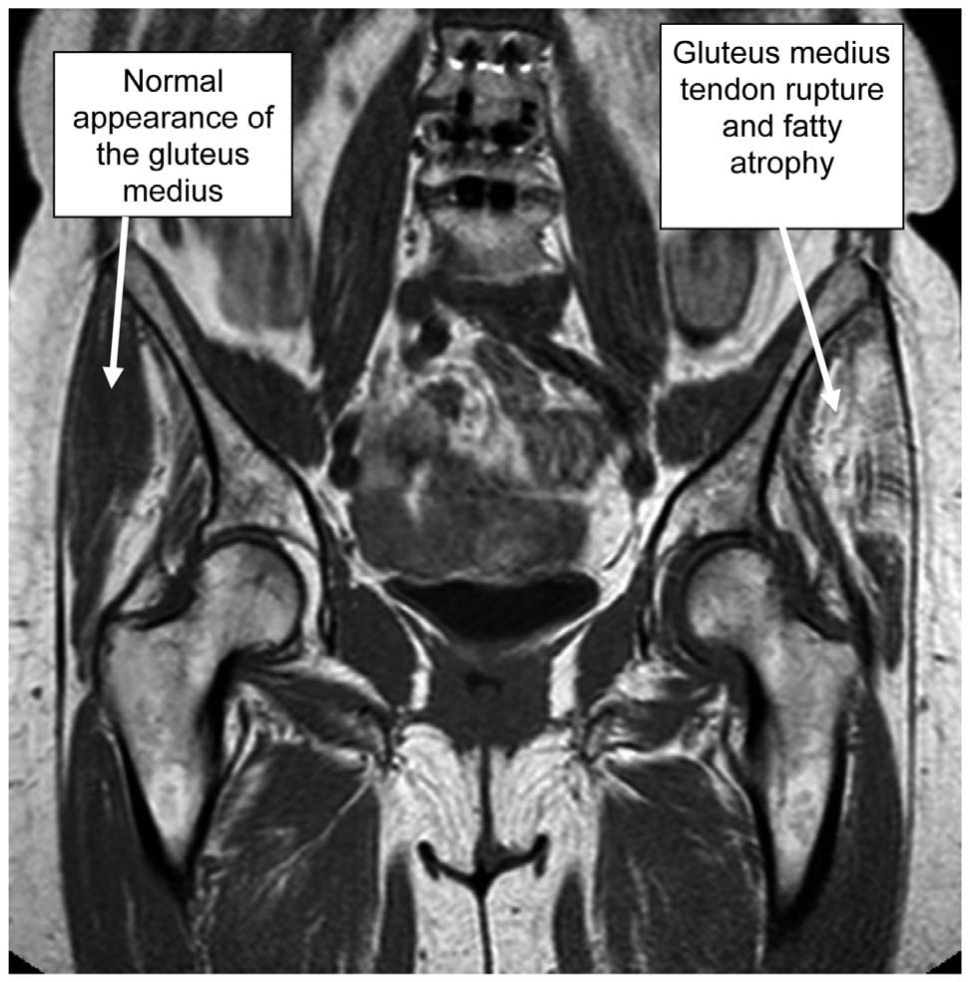
Hip abductor damage on MRI. Shown is the coronal T1-weighted MR-image at the level of the greater trochanter of a 72 year old female patient, complaining of severe lateral hip pain on the left side, radiating down to the knee. The greater trochanter is very tender on palpation and the patient presents with a reduced walking distance and difficulties during the gait cycle. The Hip Lag Sign was positive on the left side.

Hip pain represents a common cause of consultation in the orthopaedic departments around the world. Unfortunately the aetiology is often unknown or can only be confirmed by conducting expensive, time-consuming diagnostic tests, e.g. MRI (see Fig. 3). Our study demonstrates that the Hip Lag Sign is a very accurate test to predict hip abductor damage, which is one of the major causes of hip pain and greater trochanteric pain syndrome [2], [3]. It has a high sensitivity and specificity and therefore presents a viable diagnostic option that takes less than a minute and is very easy to perform while bringing no extra costs to a struggling healthcare system.All used statistical tests were able to show a significant correlation of Hip Lag Signresults and hip abductor damage (p<0.001). The Hip Lag Sign's simplicity and concurrent accuracy especially advocates its use in rural areas or developing countries where no access to further technical diagnostics such as MRI is available.

The specificity of Hip Lag Sign being 96.55% nicely demonstrates that if negative, the patient, with a high probability, does not have hip abductor damage and the physician can focus his often restricted resources on other reasons for hip pain. Moreover, a positive predictive value of 94.44% signifies that at least 94 out of 100 patients tested are true positive, meaning that they are diagnosed correctly.For these patients, inappropriate and potentially harming treatments such as corticosteroid injections should be prevented. Since tendinosis as well as partial and full thickness gluteus tendon tears can be repaired endoscopically with good results regarding pain relief and function, the Hip Lag Sign-positive patient should be transferred to an experienced hip surgeon for further therapy [17], [18].

Our findings suggest that the modified Harris Hip Score and the visual analogue scale do not correlate with the Hip Lag Sign or the MRI results in a statistically significant manner.

Strikingly, compared to the usually performed Trendelenburg test to examine the hip abductors, for which a sensitivity of only 72.7% and a specificity of only 76.9% are reported, we could show that the Hip Lag Sign is a more reliable clinical sign for this entity [19]. However, comparison of the widely used Trendelenburg's test with the new Hip Lag Sign needs to be investigated further in the future.

There are limitations ofthis study. The number of participants was rather small, and this naturally influences the statistical outcome. This also explains why the diagnostic Odds Ratio had such a wide-ranging confidence interval. Nevertheless, a diagnostic Odds Ratio of 238 means that if a patient has a positive Hip Lag Sign, the probability that he/she has hip abductor damage is significantly 238 times higher than if tested negative (p<0.001).

If this were a multi-centre, randomized controlledtrial, the number of cases would have been much higher, and the statistical analysis more precise. Thus, we suggest conducting a study with a higher evidence-level to confirm the promising results reported in this manuscript.

In conclusion, we find that the Hip Lag Sign is a very accurate, new way of looking for hip abductor damage and we recommend its inclusion into the common physical examination of the hip.
